# TP53-mediated therapy-related clonal hematopoiesis contributes to doxorubicin-induced cardiomyopathy by augmenting a neutrophil-mediated cytotoxic response

**DOI:** 10.1172/jci.insight.146076

**Published:** 2021-07-08

**Authors:** Soichi Sano, Ying Wang, Hayato Ogawa, Keita Horitani, Miho Sano, Ariel H. Polizio, Anupreet Kour, Yoshimitsu Yura, Heather Doviak, Kenneth Walsh

**Affiliations:** 1 Hematovascular Biology Center, Robert M. Berne Cardiovascular Research Center, University of Virginia School of Medicine, Charlottesville, Virginia, USA.; 2 Department of Cardiology, Osaka City University Graduate School of Medicine, Osaka, Japan.

**Keywords:** Cardiology, Cardiovascular disease

## Abstract

Therapy-related clonal hematopoiesis (t-CH) is often observed in cancer survivors. This form of clonal hematopoiesis typically involves somatic mutations in driver genes that encode components of the DNA damage response and confer hematopoietic stem and progenitor cells (HSPCs) with resistance to the genotoxic stress of the cancer therapy. Here, we established a model of TP53-mediated t-CH through the transfer of Trp53 mutant HSPCs to mice, followed by treatment with a course of the chemotherapeutic agent doxorubicin. These studies revealed that neutrophil infiltration in the heart significantly contributes to doxorubicin-induced cardiac toxicity and that this condition is amplified in the model of *Trp53*-mediated t-CH. These data suggest that t-CH could contribute to the elevated heart failure risk that occurs in cancer survivors who have been treated with genotoxic agents.

## Introduction

Anthracyclines, such as doxorubicin (Dox), are an essential component of many treatment regimens for both solid and hematologic cancers. However, the clinical utility of these drugs is limited by their cardiotoxicity. It is estimated that as many as 10% of patients treated with anthracyclines will develop some form of cardiotoxicity (1–3). Long-term anthracycline cardiotoxicity can occur within 1 year of therapy or can develop years after therapy completion (4), with more recent data suggesting that the early- and late-onset disease may represent different stages of the same evolving phenomenon (5). Regardless, mortality due to cardiovascular disease (CVD) in cancer survivors is typically greater than that due to cancer itself after a 10-year follow-up (6), and premature CVD is the leading cause of death among aging people who were treated for cancer as children (7, 8). This chronic cardiotoxicity is considered to be irreversible, refractory to standard heart failure therapy, and associated with very poor prognosis. Thus, there is an obvious need to understand this time-dependent cardiotoxicity and to overcome these hurdles.

Clonal hematopoiesis in the absence of overt hematological abnormalities is caused by precancerous clonal expansions in hematopoietic stem and progenitor cells (HSPCs). These clonal expansions can result from somatic mutations in specific driver genes that confer a fitness advantage to the cell (9, 10). These mutations are expressed in the progeny leukocytes, contributing to inflammatory processes that appear to promote mortality and morbidity. Age-related clonal hematopoiesis (ARCH) is prevalent in elderly individuals, and it is characterized by somatic mutations in *TET2*, *DNMT3A*, and other genes and associated with an increased risk of CVD and poor CVD prognosis (11–13). We and others have provided experimental evidence that various forms of ARCH can modulate inflammatory responses and contribute to CVD pathology in a gene-specific manner (13–19). In contrast to ARCH, therapy-related clonal hematopoiesis (t-CH) is an aggressive form of clonal hematopoiesis that occurs in individuals who have undergone oncological therapies (20, 21). t-CH is typically associated with genes that participate in the DNA damage response (DDR), such as *TP53* and *PPM1D*, that confer cellular resistance to genotoxic stress. Studies indicate that *TP53* and *PPM1D* mutant HSPCs preexist as small clones that undergo rapid clonal expansion in response to the cytotoxic therapy, likely due to the survival advantage against genotoxic stress that the mutation confers upon the stem cell (22–25). Whether t-CH is associated with an increased risk of chemotherapy-induced CVD is unknown.

## Results

**Figure 1:**
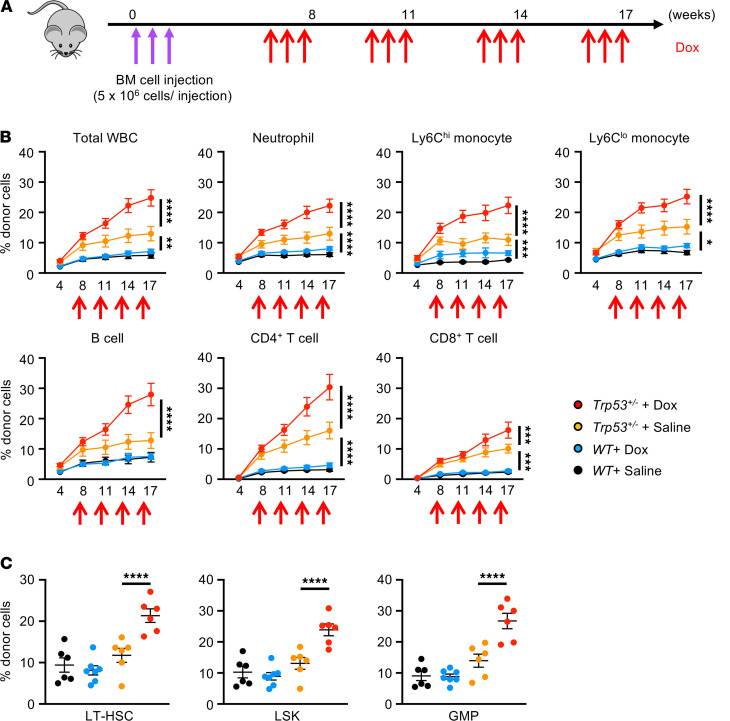
Dox treatment promotes the expansion of *Trp53* mutant hematopoietic cells in blood and bone marrow. (**A**) Schematic of adoptive bone marrow transplantation and Dox administration. A total of 1.5 *×* 10^7^ unfractionated donor bone marrow cells were sequentially injected into nonconditioned B6.SJL-CD45.1 recipients over 3 consecutive days (indicated by purple arrows). Donor cells were obtained from either C57BL/6J wild-type mice (*Trp53^+/+^*) or *Trp53* heterozygous–deficient (*Trp53^+/–^*) mice. Recipient mice were injected with Dox at 7 weeks after BMT involving 4 rounds of 6 μg/g i.p. injection (2 μg/g/d over 3 consecutive days), with 3 weeks between each round (indicated by red arrows). Absolute number and the chimerism of test cells in peripheral blood at baseline and after each cycle of Dox or saline administration were evaluated by sequential flow cytometry analysis. (**B**) Flow cytometry analysis of blood chimerism over the time course to show the progressive expansion of *Trp53^+/–^* mutant clones in total WBCs, neutrophils, Ly6C^hi^ monocytes, Ly6C^lo^ monocytes, B cells, CD4^+^ T cells, and CD8^+^ T cells after Dox treatment compared with saline administration (*n* = 6–7 per group). The approximate times of multiple Dox injections are indicated. Statistical analysis was performed with 2-way ANOVA with Tukey’s multiple-comparison tests. (**C**) Flow cytometry analysis of bone marrow at 19 weeks after adoptive BMT showing increased chimerism (percentage) of donor-derived LT-HSCs, LSKs, and GMPs in mice transplanted with *Trp53* heterozygous–deficient cells compared with wild-type cells after 4 rounds of Dox or saline treatment (*n* = 6–7 per group). Statistical analysis was performed with 2-way ANOVA with Tukey’s multiple comparisons tests. *P < 0.05, **P < 0.01, ***P < 0.001, ****P < 0.0001. LT-HSC, long-term hematopoietic stem cell; LSK, lineage^–^Sca1^+^C-kit^+^ cell; GMP, granulocyte-monocyte progenitor.

We established the animal model of clonal hematopoiesis in which a small proportion of HSPCs harbor the *Trp53* mutation. To overcome the possible limitations of myeloablative bone marrow transplant (BMT), we initially employed an adoptive transfer technique in which total bone marrow cells were injected to nonirradiated mice (Figure 1A) (14, 26). In this model, *Trp53* heterozygous–deficient (*Trp53*^+/–^) mice were used as donors to avoid hematologic malignancy and recapitulate the clinical state of clonal hematopoiesis in which 1 allele is typically mutated. Successful engraftment leading to the time-dependent expansion of the mutant HSPCs in the unchallenged mice was achieved when a total of 1.5 × 10^7^ unfractionated BM cells was transferred intravenously to each recipient mouse on 3 consecutive days (Figure 1B). Mice were then treated with Dox or saline as control, administered in cycles, to examine the competitive fitness of *Trp53*^+/–^ HSPCs under the conditions of chemotherapeutic stress. The analysis of total white blood cells, neutrophils, and Ly6C^hi^ monocytes in the control condition (saline) revealed that donor-derived *Trp53*^+/–^ cells achieved significantly higher levels of chimerism compared with wild-type cells over the 4-month time course, indicating a fitness advantage of *Trp53*^+/–^ clones under homeostatic conditions (Figure 1B). In support of the concept that *TP53* is a driver of t-CH, Dox administration resulted in the further expansion of *Trp53*^+/–^ cells but had little or no effect on the minimal expansion of transplanted wild-type cells when circulating cell populations were assessed (Figure 1B). In agreement with the observations in circulating leukocyte populations, the proportions of donor-derived *Trp53*^+/–^ cells in bone marrow lineage^–^Sca1^+^C-kit^+^ cell, lineage^–^Sca1^+^cKit^+^CD48^-^CD150^+^ cells (long-term hematopoietic stem cell), and lineage^–^Sca1^-^cKit^+^CD16/32^hi^CD34^+^ cell (granulocyte-monocyte progenitor) fractions were considerably higher under conditions of Dox treatment compared with the saline control at the termination of the experiment (Figure 1C). Consistent with the paradigm of clonal hematopoiesis (27), there was no *Trp53*-dependent difference in hemoglobin levels or the absolute numbers of any hematopoietic cell type, although the prolonged administration of Dox altered some of these parameters in both genotypes (Supplemental Figure 1; supplemental material available online with this article; https://doi.org/10.1172/jci.insight.146076DS1).

**Figure 2:**
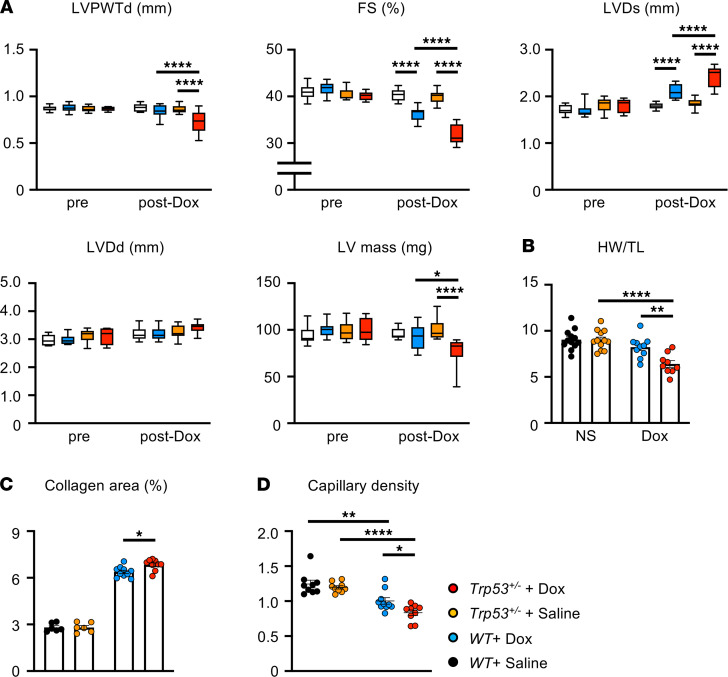
Dox-mediated acceleration of *Trp53* mutant clones promotes doxorubicin-related cardiac toxicity. (**A**) Echocardiographic analyses of fractional shortening (FS; percentage), left ventricular posterior wall thickness diameter (LVPWTd; mm), left ventricular end-systolic diameter (LVD; mm), left ventricular end-diastolic diameter (LVDd; mm), and left ventricular mass (LV mass; mg) of mice transplanted with wild-type cells or *Trp53*-insufficient cells at baseline and after 4 cycles of Dox or saline administration (*n* = 9–12 per group). Statistical analysis was performed with 2-way ANOVA with Tukey’s multiple-comparison tests. (**B**) Heart weight (HW) was adjusted by tibia length (TL) (*n* = 9–12 per group) at the end of study. (**C**) Collagen area of the heart was measured by Sirius Red/Fast Green (MilliporeSigma) staining at the end of study. (**D**) Capillary density of the heart was measured by isolectin B4 staining. Statistical analysis was performed with 2-way ANOVA with Tukey’s multiple-comparison tests. *P < 0.05, **P < 0.01, ****P < 0.0001.

To test whether the Dox-induced clonal expansion of *Trp53* mutants participates in cardiac damage, we accessed cardiac function of these animals by echocardiography over the time course of the study. While mice transplanted with wild-type cells showed Dox-dependent deterioration of cardiac contractility and a thinning of the ventricular wall, mice with the expanding *Trp53*^+/–^ clones displayed significantly greater functional impairment and wall thinning (Figure 2, A and B). Mice treated with Dox also displayed greater fibrosis and a reduced capillary density in the myocardium, and mice receiving the adoptive transfer of *Trp53*^+/–^ cells displayed a modestly augmented pathological response when assessed for these parameters (Figure 2, C and D).

The vast majority of missense *TP53* mutations in humans are mapped to its DNA-binding domain, and it has been suggested that mutants in this domain abrogate its sequence-specific DNA-binding activity (20, 21, 28). Thus, to recapitulate *TP53*-mediated clonal hematopoiesis under these conditions, we also analyzed the adoptive transfer bone marrow cells carrying the *Trp53^R270H^* mutation (equivalent to the hotspot mutation R273H located at the DNA-binding domain of human TP53). Donor-derived *Trp53^R270H^* clones exhibited a selective expansion in response to serial Dox administration, as seen in circulating leukocyte populations and bone marrow HSPC fractions (Supplemental Figure 2, A and B). Mice harboring the expanding *Trp53^R270H^* mutant blood cells displayed a significant reduction in cardiac contractility as well as a wall thinning in response to the Dox therapy regimen when compared with mice transplanted with wild-type cells (Supplemental Figure 3). While these experiments did not involve a tumor-bearing model, the collective results in the nonmyeloablative BMT mice provide evidence that *TP53*-mediated t-CH can promote Dox-induced cardiotoxicity.

To obtain additional insights, we employed a myeloablative BMT strategy using donor wild-type or *Trp53*^+/–^ bone marrow cells adjusted to a variant allele fraction (VAF) of 0.15, which is typically observed in patient cohorts with t-CH (20, 21, 24). Echocardiographic measurements revealed that, while there were no differences between *Trp53*^+/–^ and wild-type groups before treatment, significant reductions in fractional shortening and posterior wall thickness of the heart were observed after the administration of Dox (Supplemental Figure 4A). Consistent with these data, histological measurements revealed reduced cardiomyocyte size, greater myocardial fibrosis, and diminished capillary density in the group with the *Trp53* mutant cell condition (Supplemental Figure 4, B–D). Transcript analysis of hearts at 8 weeks after Dox treatment showed a significant increase in inflammatory mediators, including *Il1b*, *Il6*, and *Tnf*, in mice transplanted with mutant *Trp53* versus wild-type cells (Supplemental Figure 4E), suggesting that the *Trp53*^+/–^ mutant clones could be contributing to cardiotoxicity through an inflammatory mechanism.

**Figure 3:**
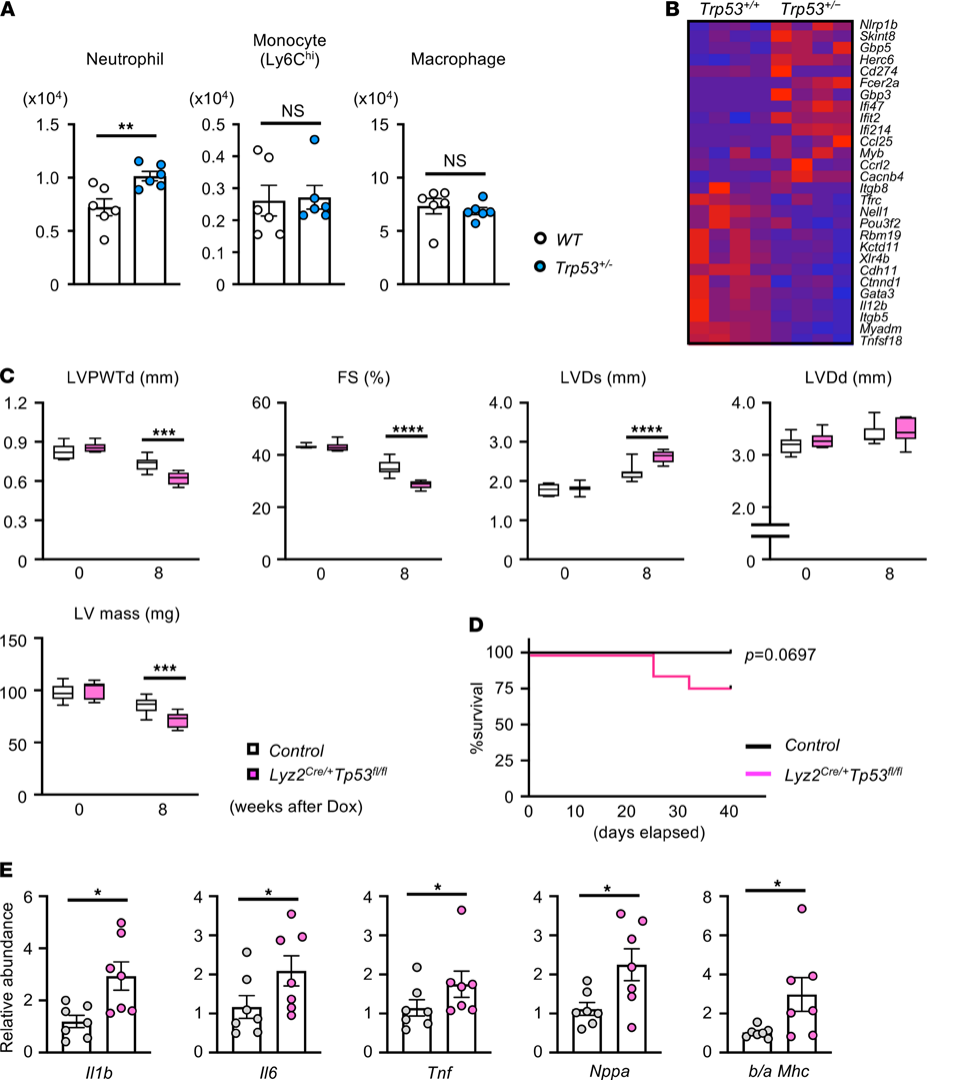
*Trp53*-deficient myeloid cells accelerate Dox-induced cardiotoxicity. (**A**) Flow cytometry analysis of cardiac immune cells from mice that underwent competitive BMT and Dox administration. In this study, mice underwent partial (30%) bone marrow reconstitution with *Trp53*-deficient cells (30% Het BMT mice) or WT cells (30% WT BMT mice). After a 7-week recovery from BMT, mice were injected intraperitoneally with Dox (single injection of 15 μg/g), and hearts were harvested 7 days later for flow cytometry analysis of resident immune cells (*n* = 6). Statistical analysis was performed with unpaired (2-tailed) Student’s *t* test (neutrophil) and Mann-Whitney *U* tests (Ly6C^hi^ monocyte, macrophage). (**B**) Heatmap of select group of the most highly differentially expressed genes from the highest ranked annotation categories comparing blood neutrophils between *Trp53*-sufficient (*Trp53^+/+^*) and *Trp53* heterozygous–deficient mice (*Trp53^+/–^*) at 1 day after Dox administration (15 μg/g single injection). Mice were administered Dox by intraperitoneal injection (15 mg/kg split into 3 injections over 10 days). (**C**) Echocardiographic analysis of left ventricular posterior wall thickness diameter (LVPWTd; mm), fractional shortening (FS; %), left ventricular end-systolic diameter (LVDs; mm), left ventricular end-diastolic diameter (LVDd; mm), and left ventricular mass (LV mass; mg) of myeloid-specific *Trp53*-deficient (*Lyz2^Cre/+^Tp53^fl/f^*) mice and littermate controls (Control) before and at 8 weeks after Dox administration. Statistical analysis was performed with 2-way repeated-measures ANOVA with Sidak’s multiple-comparisons tests (*n* = 7 per group). (**D**) Survival of myeloid-specific *Trp53*-deficient mice and littermate controls after Dox administration. Survival curve was obtained by the Kaplan-Meier method. Statistical analysis was performed with log-rank test (*n* = 12 per genotype). (**E**) Real-time qPCR analysis of transcript expression in myocardium obtained from *Lyz2*^Cre/+^*Trp53*^fl/fl^ and littermate control mice at 8 weeks after Dox treatment (*n* = 7 per genotype). Statistical analysis was performed with unpaired (2-tailed) Student’s *t* test (*Il6, Il1b, b/a MHC*), 2-tailed unpaired Student’s *t* test with Welch correction (*Nppa*), or Mann-Whitney *U* test (*Tnf*). *P < 0.05, **P < 0.01, ***P < 0.001, ****P < 0.0001.

To further define the role of the immune system in Dox-mediated cardiotoxicity, wild-type mice were administered a single bolus of Dox, and cardiac parameters, peripheral blood cell counts, and levels of cardiac-resident leukocytes were assessed at different time points (Supplemental Figure 5A). In this model, Dox administration led to the time-dependent loss of body weight and heart weight and to reductions in cardiac wall dimension and function (Supplemental Figure 5, B–D). These effects were accompanied by a transient increase of peripheral blood monocytes at the 7-day time point and an increase in neutrophils at 7 days that was sustained until the termination of the experiment at 14 days (Supplemental Figure 5E). The analysis of cardiac-resident leukocytes revealed that monocyte number increased while macrophage number decreased, with a peak and nadir at the 7-day time point, respectively (Supplemental Figure 6). Notably, Dox-induced neutrophil recruitment to the heart reached a peak at 7 days, and levels were maintained until the termination of the experiment (Supplemental Figure 6). This behavior contrasts with other models of cardiac injury that display a much more rapid and transient influx of neutrophils (29, 30). Furthermore, mice that had been transplanted with *Trp53* heterozygous mutant bone marrow cells displayed greater neutrophil recruitment at the 7-day time point compared with mice transplanted with wild-type cells, but treatment with Dox had no effect on the numbers of Ly6C^hi^ monocytes or macrophages within cardiac tissue under these conditions (Figure 3A).

Recent studies have challenged the long-held view that neutrophils are transcriptionally silent (31). Thus, to evaluate the effect of *Trp53* deficiency on neutrophil phenotype, RNA sequence analysis was performed on peripheral blood neutrophils from *Trp53*^+/–^ and wild-type mice at 1 day after Dox administration (Supplemental Table 1). Principal component analysis revealed that *Trp53*^+/–^ neutrophils have a gene expression profile distinct from that of wild-type neutrophils (Supplemental Figure 7A). With a cut off value of *P* < 0.05 and TPM fold change of >1.5, 898 genes were overexpressed and 63 genes were underexpressed in *Trp53*^+/–^ neutrophils (Supplemental Figure 7B). Genes linked to immune response and cytokine response were significantly enriched in *Trp53*^+/–^ neutrophils, among which were genes related to the inflammasome pathway (*Nlrp1b*, *Gbp5*, *Il18*) and chemokines, such as *Ccl25,* *Ccrl2*, and *Cxcl1* (Figure 3B; Supplemental Table 2).

In view of these findings, experiments were performed to assess the roles of different myeloid cell populations in Dox-induced cardiac toxicity. *Ccr2*-deficient mice exhibit defects in monocyte egress from bone marrow (32). Consistently, the myocardium of Ccr2-deficient mice was largely devoid of CCR2^+^-infiltrating monocyte/macrophages compared with wild-type mice, but neutrophil content was not altered (Supplemental Figure 8A). Notably, *Ccr2*-deficient mice displayed comparable cardiac dysfunction after treatment with Dox relative to wild-type mice, suggesting little or no contribution of monocytes/macrophages to Dox-induced cardiac toxicity (Supplemental Figure 8B). To examine the consequences of neutrophil infiltration in this model, wild-type mice were treated with Ly6G antibody to deplete neutrophils and Dox-induced cardiac toxicity was assessed. The Ly6G antibody largely protected against cardiac dysfunction, as assessed by measures of posterior wall dimension and fractional shortening (Supplemental Figure 9A). Treatment with the Ly6G antibody also diminished the expression of the heart failure marker *Nppa* and the ratio of *Mhc* isoforms (Supplemental Figure 9B), and it reduced the level of oxidative stress in the myocardium, as assessed by the accumulation of nitrotyrosine-protein adducts (Supplemental Figure 9C). In support of these findings, treatment with the pharmacological CXCR2 inhibitor SB265610, which functions to inhibit neutrophil trafficking, reduced neutrophil influx to the hearts of Dox-treated mice (Supplemental Figure 10A) and reversed the effects of Dox on cardiac wall thinning and function (Supplemental Figure 10B). Collectively, these data suggest that neutrophil involvement is a significant component of Dox-mediated cardiac toxicity in wild-type mice.

Further experiments examined myeloid cell participation in the amplified cardiac toxicity observed in the *Trp53*-mediated t-CH model. To this end, myeloid-specific *Trp53*-deficient (*Lyz2^Cre/+^Trp53^fl/fl^*) mice treated with Dox displayed marked reduction in cardiac function compared with control (*Lyz2^+/+^Trp53^fl/fl^*) mice (Figure 3C). Notably, the Dox-induced cardiac toxicity was much more severe in this strain than in mice with partial *Trp53* deficiency (compare with Supplemental Figure 4), and this severe dysfunction was associated with a trend toward reduced survival compared with that of control mice treated with Dox (Figure 3D). The myeloid-restricted *Trp53*-deficient mice also displayed upregulation of transcripts that encode inflammatory mediators in the myocardium compared with control mice (Figure 3E).

**Figure 4:**
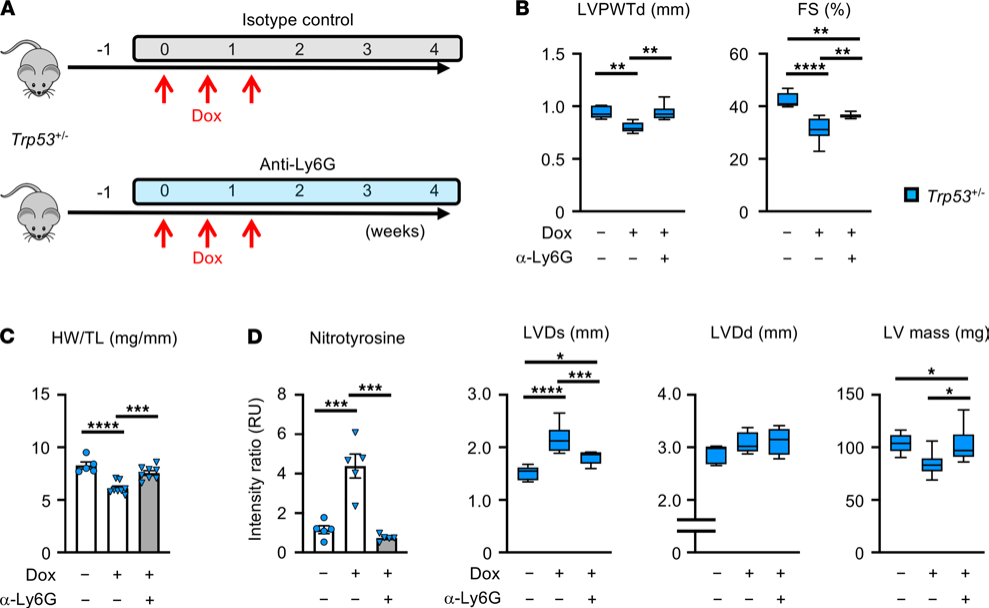
Neutrophil overactivation contributes to the cardiac toxicity induced by *Trp53*-mediated clonal hematopoiesis. (**A**) Schematic of the study in **B**–**D**. Lethally irradiated mice underwent a competitive BMT with 30% *Trp53* heterozygous–deficient (*Trp53^+/–^*) cells. After a 6-week recovery from BMT, mice received an intraperitoneal administration of anti-Ly6G (clone 1A8, neutrophil depletion) or isotype control (clone 2A3) antibody (antibody, 500 μg/injection/mouse, 1 injection/3 days for 4 weeks) from 1 day before Dox administration (15 μg/g, split into 3 injections over 10 days) until the end of this study. (**B**) Left ventricular posterior wall thickness diameter (LVPWTd; mm), fractional shortening (FS; %), left ventricular end-systolic diameter (LVDs; mm), left ventricular end-diastolic diameter (LVDd; mm), and left ventricular mass (LV mass; mg) at the end of study. (**C**) Heart weight (HW) adjusted to tibia length (TL). (**D**) Relative immunofluorescence values of 3-nitrotyrosine staining in heart sections of mice. All mice were treated with sterile saline, Dox + isotype control, or Dox + anti-Ly6G. Statistical analysis was performed with 2-way ANOVA with Tukey’s multiple-comparison tests. For **B**–**D**, *n* = 5–8 per group. *P < 0.05, **P < 0.01, ***P < 0.001, ****P < 0.0001.

To investigate whether neutrophil involvement is essential for cardiac toxicity in the t-CH model, mice underwent BMT to establish a VAF of 0.15 with heterozygous Trp53-deficient cells and were then treated with a course of Dox or saline in the presence of anti-Ly6G antibody, to deplete neutrophils, or an isotype control antibody (Figure 4A). Neutrophil depletion ameliorated the detrimental effects of Dox on heart weight and echocardiographic parameters in mice with the expanding *Trp53*-deficient HSPCs (Figure 4, B and C). Assessing nitrotyrosine-protein adducts in myocardial tissues from the different experimental groups of mice revealed that the *Trp53*-deficient condition markedly augmented nitrotyrosine content and that neutrophil depletion with anti-Ly6G antibody reversed this damage (Figure 4D).

## Discussion

Dox and other anthracyclines are used to treat many solid and hematologic cancers. However, the clinical utility of this class of drugs is limited by their immediate and prolonged cardiac toxicity (1, 3). Studies of anthracycline effects on the heart have largely focused on cardiac myocyte toxicity (33, 34), while the potential role of immune cells in this condition has received less attention. The data from the current study reveal that Dox-induced cardiotoxicity involves an unexpected and complex interplay between the myocardium and the immune system. It has previously been shown that Dox interacts with topoisomerase II β, leading to DNA double-strand breaks, mitochondrial dysfunction, elevations in oxidative and nitrosative stress, and death in cardiac myocytes (35). Here, we report that Dox therapy will lead to the prolonged infiltration of neutrophils to the heart and that neutrophil depletion or inhibition will diminish the cardiotoxic actions of Dox. Oxidative/nitrosative stress damage is a widely recognized component of Dox cardiac toxicity, and the experimental data provided herein suggest that infiltrating neutrophils are a significant source of this stress. Consistent with these experimental findings, clinical studies have found that anthracycline-induced heart failure is associated with elevated serum levels of neutrophil marker proteins and genetic variants that lead to neutrophil ROS/reactive nitrogen species generation (36–38). While traditionally thought to largely participate in acute inflammatory responses, these data add to the growing realization that neutrophils can contribute to chronic disease processes (31, 39).

This study also provides evidence to suggest that t-CH is a factor that contributes to the cardiac toxicity that develops in patients with cancer who are treated with anthracyclines and that this effect operates, at least in part, by augmenting the infiltration and activation of neutrophils. Specifically, we have shown that Trp53-deficient HSPCs undergo rapid expansion in response to Dox treatment and that the mutant neutrophil progeny of these HSPCs markedly augment the cardiotoxicity that results from Dox therapy. In Dox-treated mice, we found that heterozygous Trp53 deficiency in neutrophils leads to further elevations in myocardial oxidative/nitrosative stress and that Trp53-deficient neutrophils display numerous transcriptional changes, including the elevation of chemokine transcripts that promote neutrophil recruitment and activation. In light of these data, it is tempting to speculate that t-CH can impact both the medium- and long-term effects of anthracyclines on the heart. As indicated by the experimental models, Dox administration can lead to the rapid expansion of a preexisting TP53 clone that amplifies the cardiotoxicity of the Dox therapy. Furthermore, once these clones have undergone expansion, they can potentially exert chronic pathological actions in the myocardium of aging individuals who have been treated for cancer when they are exposed to other cardiovascular stresses. Collectively, these data suggest that t-CH can be predictive of heart failure in people who have been treated for cancer and that assessing the status of clonal hematopoiesis before and after cancer therapy could provide guidance for personalized therapies to protect the heart from the chronic effects of genotoxic drugs. Future investigations using samples from patients with Dox-induced cardiotoxicity are warranted.

## Methods

Additional descriptions are included in the Supplemental Methods.

### Mice

Wild-type mice (*Cd45.2*), *Trp53*-insufficient mice, *Trp53^R270H^* mice, *Trp53*-floxed mice, *Lyz2-Cre* mice, Pep Boy mice (*Cd45.1*), and *Ccr2^gfp/gfp^* mice were obtained from The Jackson Laboratory (stock 000664, 002103, 008182, 008462, 004781, 002014, and 027619, respectively). All strains were on a C57BL/6J background. Mice with myeloid-restricted *Trp53* ablation were generated by crossing *Trp53*-floxed mice (*Trp53^fl/fl^*) with *Lyz2-Cre* mice. Male mice were used for both in vivo and in vitro experiments unless otherwise noted. Mice were housed in a specific pathogen–free animal facility and given food and water ad libitum on 12-hour-dark/light schedule.

### Bone marrow transplantation

#### Nonmyeloablative bone marrow transplantation.

Eight- to twelve-week-old C57BL/6J *Cd45.1* Pep Boy mice were transplanted with suspensions of bone marrow cells from two patterns of donors: (a) either *Trp53^+/–^* or wild-type (*Trp53^+/+^*) mice and (b) either *Trp53^R270H^* or wild-type (*Trp53^R270H/+^* and *Trp53^+/+^*) mice. 5 *×* 10^6^ of unfractionated bone marrow cells were injected to nonirradiated recipients via retro-orbital vein over consecutive 3 days (1.5 *×* 10^7^ cells in total) as previously described (25).

#### Myeloablative bone marrow transplantation.

Eight- to twelve-week-old lethally irradiated C57BL/6 *Cd45.1* Pep Boy recipients were transplanted with suspensions of bone marrow cells containing 30% *Cd45.2 Trp53^+/–^* cells and 70% *Cd45.1 Trp53^+/+^* cells (30% *Het* mice) or 30% *Cd45.2 Trp53^+/+^* cells and 70% *Cd45.1 Trp53^+/+^* cells (30% wild-type mice). Bone marrow cells were isolated from femurs and tibias of donor 8- to 12-week-old mice after euthanasia. Recipient mice were irradiated in a pie cage (Braintree Scientific, catalog IRD-P R) to limit mobility and ensure an equal dose of irradiation and were exposed to 2 radiation doses of 5.5 Gy with 4 hours apart using a Cesium irradiator (11 Gy in total). After the second irradiation, each recipient mouse was injected with 5 *×* 10^6^ bone marrow cells via the retro-orbital vein plexus. Sterilized caging, food, and water were provided during the first 14 days after transplantation, and water was supplemented with the antibiotic (Sulfatrim, Teva, catalog 00703-9526-01).

### Doxorubicin administration

#### Pattern 1.

Adoptive BMT mice were injected with doxorubicin 6 weeks after BMT using a model of therapy-related clonal hematopoiesis (24) involving 3 rounds of 2 mg/kg i.p. injection, with 3 weeks between each round.

#### Pattern 2.

Competitive BMT mice, *Trp53-Myelo*–KO mice, *Ccr2^gfp/gfp^* mice, and mice in neutrophil depletion and neutrophil recruitment inhibition studies were injected with doxorubicin at doses of 15 μg/g (body weight) split into 3 injections at indicated time points.

#### Pattern 3.

Wild-type mice were injected with a single dose of doxorubicin (15 μg/g) at indicated time points.

### Monoclonal anti-mouse Ly6G antibody administration

This antibody was used to deplete neutrophils in vivo as previously described (29). Mice were injected intraperitoneally with 500 μg/injection of antibody every 3 days over a time course starting from 2 days before the start of doxorubicin administration until the end of the study as described previously (29). Isotype control anti-Trinitrophenol antibody (rat IgG2a) was similarly injected into a control group of mice. Antibody against Ly6G (clone 1A8) and rat IgG2 control antibody (clone 2A3) were purchased from BioXcell (catalog BP0075-1 and BP0089, respectively). These antibodies were stored at 4°C, and the solutions were mixed with PBS before injection (total volume of 200 μL). Injections were performed using a sterile syringe and 29-gauge needle. The expiration date of the mixtures coincided with the expiration date of the antibodies.

### Statistics

GraphPad Prism 8.0 was used for statistical analyses of all the experiments. Data are shown as mean ± SEM, except for in the box plots, in which the whiskers extend from minimum to maximum. The Shapiro-Wilk normality test was used to analyze data normality. Statistical tests included unpaired, 2-tailed Student’s *t* test (with Welch correction when variance was unequal) for normally distributed data and Mann-Whitney *U* test for nonnormally distributed data. For multiple comparisons, 1-way ANOVA with post hoc Tukey’s test (normally distributed data) or Kruskal-Wallis H test with post hoc Dunn’s test (nonnormal distributed data) was performed. Data with more than 1 variable were evaluated by 2-way ANOVA with post hoc Tukey’s tests. Sequential data were evaluated by 2-way repeated-measures ANOVA with post hoc Sidak or Tukey’s multiple-comparison tests. *P* values were considered significantly different at 0.05.

### Study approval

All procedures involving animals were approved by the Institutional Animal Care and Use Committee at the University of Virginia.

## Author contributions

SS and KW designed the project. SS, YW, HO, KH, MS, YY, AHP, AK, and HD performed experiments. SS, YW, HO, and KW prepared the manuscript. All authors approved the manuscript.

## Supplementary Material

Supplemental data

## Version 1 07/08/2021

Electronic publication
