# Supplementary material for: TP53-mediated therapy-related clonal hematopoiesis contributes to doxorubicin-induced cardiomyopathy by augmenting a neutrophil-mediated cytotoxic response
Source: JCI Insight. 2021 Jul 8;6(13):e146076. doi: 10.1172/jci.insight.146076 (PMC8410064; doi:10.1172/jci.insight.146076)
Supplement: Supplemental data [file jciinsight-6-146076-s102.pdf]

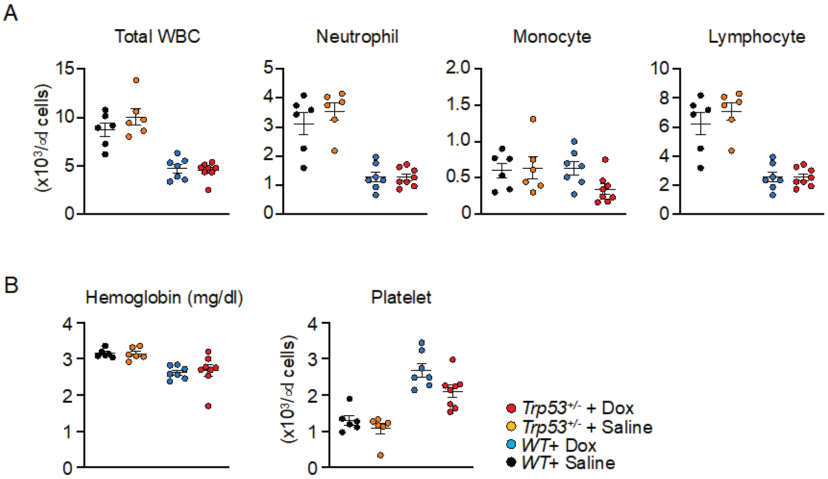

Supplemental Figure 1. *Trp53*-mediated cell expansion is not associated with changes in absolute blood cell number. A. Analysis of peripheral blood cell numbers of mice in different experimental groups: wild type (WT) + Saline in black, WT + Dox in blue, *Trp53*$^{+/-}$ + Saline in yellow, *Trp53*$^{+/-}$ + Dox in red. **B**. Hemoglobin and platelet levels in the same experimental groups. Statistical analysis was performed with 2-way ANOVA with Tukey's multiple-comparisons tests. (n=6-8 per group). Cell numbers were analyzed after the fourth cycle of Dox or saline treatment.

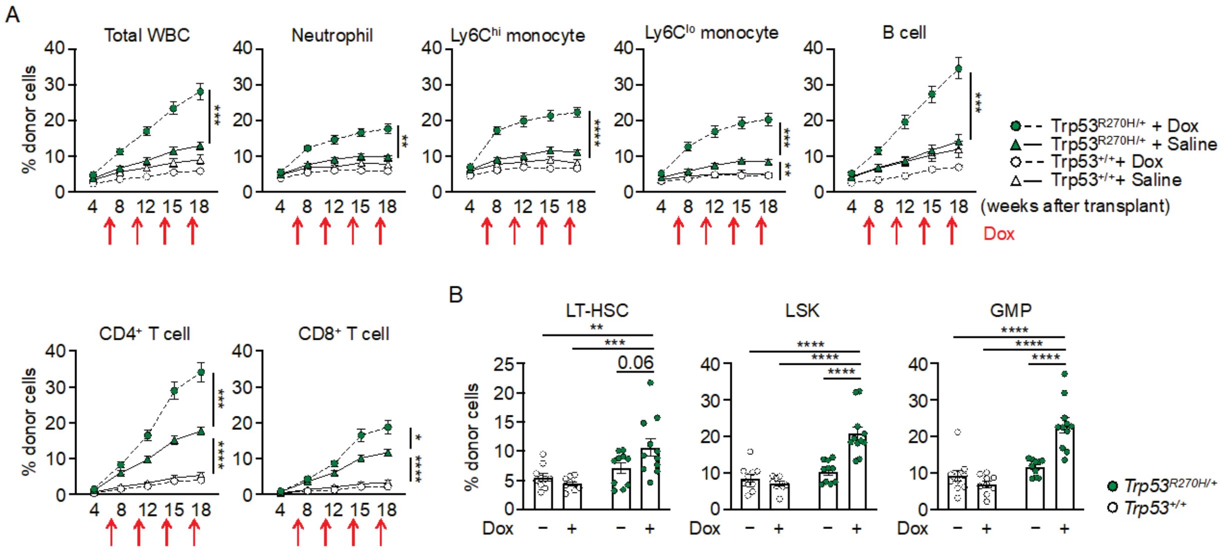

Supplemental Figure 2. Dox exposure promotes the expansion of *Trp53^{R270H}* mutant cells in hematopoietic stem and progenitor cells in the non-myeloablative bone marrow transplantation model. A. Flow cytometry analysis of blood chimerism over the time reveals the progressive expansion of *Trp53^{R270H}* mutant clones in total WBC, neutrophil, Ly6C^{hi} monocyte, Ly6C^{lo} monocytes, B cells, CD4^{+}T cells and CD8^{+}T cells after 4 rounds of Dox compared to saline treatment (2 mg/kg/day over 3 consecutive days per round, with 3 weeks' interval between each round). The timing of Dox/saline administration is indicated by arrows. **B.** Flow cytometry analysis of bone marrow at 20 weeks after adoptive BMT showing increased chimerism (%) of donor-derived LSK and GMP, and an increase trend of LT-HSC in mice transplanted with *Trp53^{R270H}* vs. wild type cells after Dox. In the saline treatment condition, there was no effect on cell expansion in the *Trp53^{R270H}* vs. wild type group. n=10-11 per group. Statistical analysis was performed with 2-way repeated-measures ANOVA with Tukey's multiple-comparison tests. **p*<0.05, ***p*<0.01, ****p*<0.001, *****p*<0.0001.

LT-HSC = long-term hematopoietic stem cell; LSK = lineage^{-}Sca1^{+}C-kit^{+} cell; GMP = granulocyte-monocyte progenitor.

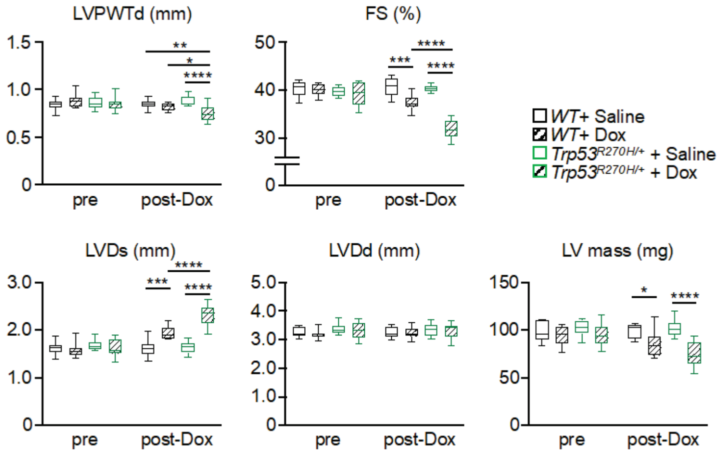

Supplemental Figure 3. The expansion of *Trp53*^{R270H} cells in blood accelerates Dox cardiotoxicity in mice. Echocardiographic analyses of left ventricular posterior wall thickness diameter (LVPWTd, mm), fractional shortening (FS, %), left ventricular end-systolic diameter (LVDs, mm), left ventricular end-diastolic diameter (LVDd, mm), and left ventricular mass (LV mass, mg) of mice transplanted with wild type cells or *Trp53*^{R270H}-mutated cells at baseline and after four cycles of Dox or saline administration. Measurements were made at the 20 week time point after adoptive BMT. Statistical analysis was performed with 2-way repeated-measures ANOVA with Tukey's multiple-comparisons tests. (n=10-11 per group). *p<0.05, **p<0.01, ***p<0.001, ****p<0.0001.

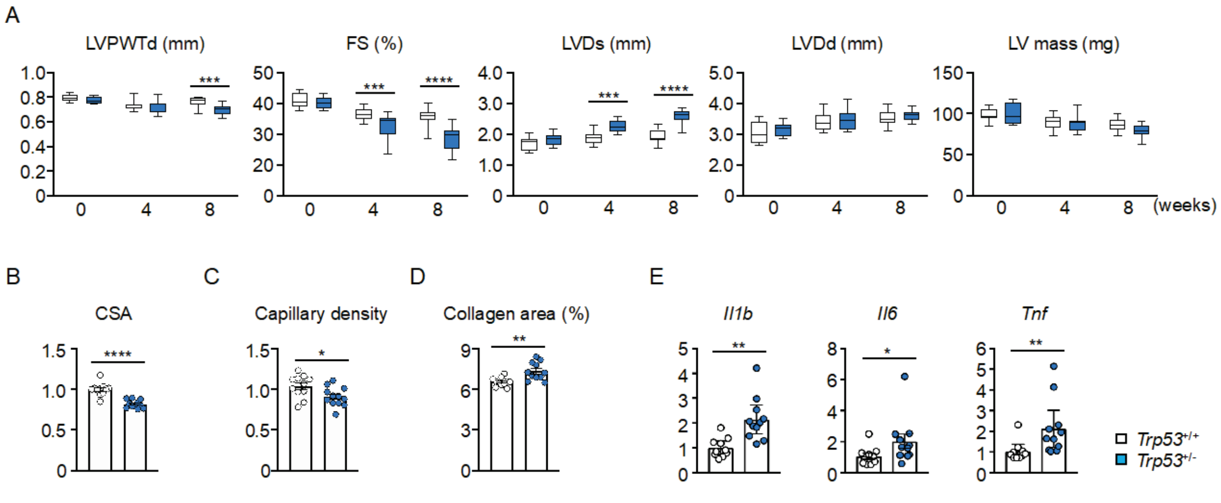

Supplemental Figure 4. *Trp53*-mutated hematopoietic cells enhance myocardial inflammation and accelerate Dox cardiotoxicity. A. Analyses of left ventricular posterior wall thickness diameter (LVPWTd, mm), fractional shortening (FS, %), left ventricular end-systolic diameter (LVDs, mm), left ventricular end-diastolic diameter (LVDd, mm), and left ventricular mass (LV mass, mg) of lethally irradiated mice transplanted with 30% wild type cells or 30% *Trp53*-heterozygous cells. After 4 weeks of recovery, mice were treated and with Dox or saline for 8 weeks. Dox or saline control was administered intraperitoneally by a sterile syringe and needle (29G) with a dose of 15 mg/kg (body weight) split into 3 injections over 10 days. Statistical analysis was performed with 2-way repeated measures ANOVA with Sidak's multiple-comparisons tests. Echocardiography was performed at indicated time points post-BMT. **B**. Quantification analysis of cross-sectional area (CSA) of cardiomyocyte by wheat germ agglutinin staining of the heart sections from each experimental group (*Trp53*$^{+/+}$ in White, *Trp53*$^{+/-}$ in Blue) at the end of the study. Statistical analysis was performed with 2-tailed unpaired Student's *t* test. **C.** Collagen area of the heart measured by Sirius Red/Fast Green staining at the end of study. **D.** Capillary density of the heart measured by isolectin B4 staining. **E**. Real time qPCR analysis of transcript expression in hearts from the different experimental groups. *36b4* was used as a reference control. Statistical analysis was performed with 2-tailed unpaired Student's *t* test with Welch correction (*Il-1b*) and Mann-Whitney U test (*Il-6, Tnf*). For **A-E**, n=10-11 per genotype. *p<0.05, **p<0.01, ***p<0.001, ****p<0.0001.

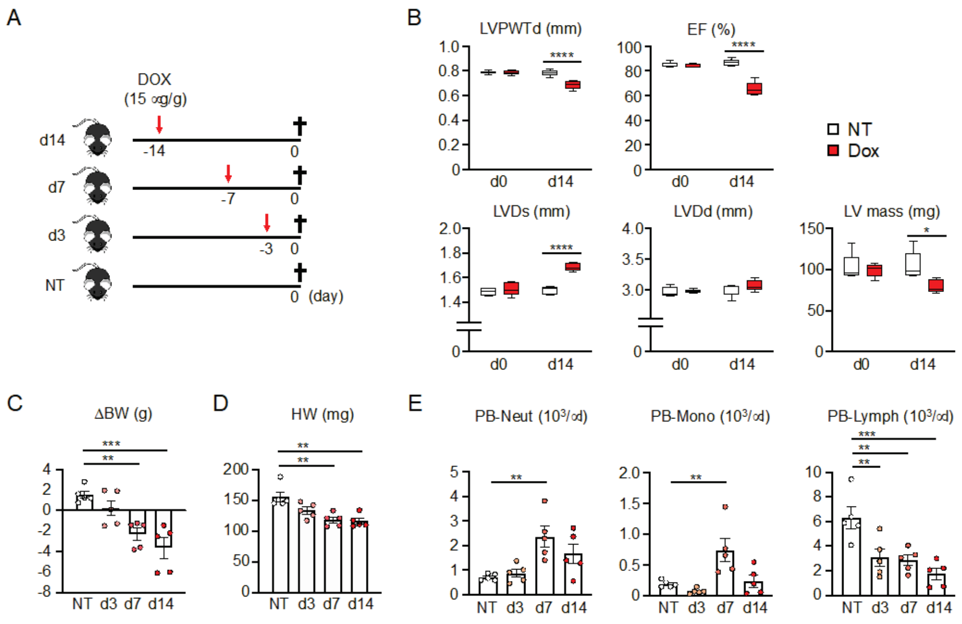

Supplemental Figure 5. Dynamic changes in cardiac immune cells following Dox administration to *wild type* mice. A. Schematic of the study. C57BL6/J mice were administered a single injection of Dox (15 mg/kg) intraperitoneally at indicated time points. Mice in non-treated (NT) group were injected with sterile saline. **B.** Echocardiographic analysis of left ventricular posterior wall thickness diameter (LVPWTd, mm), fractional shortening (FS, %), left ventricular end-systolic diameter (LVDs, mm), left ventricular end-diastolic diameter (LVDd, mm), and left ventricular mass (LV mass, mg) of mice at baseline and 14 days post-Dox or saline administration (n=4). Statistical analysis was performed with 2-way repeated-measures ANOVA with Sidak's multiple-comparisons tests. **C.** Body weight (BW, g) change of mice from each group at the end of study (n=5 per time point). **D.** Heart weight (HW, mg) of mice from each group at the end of study (n=5 per time point). Statistical analysis was performed with Kruskal-Wallis H test with Dunn's multiple-comparisons tests. **E.** Analysis of peripheral blood (PB) neutrophil (Neut), monocytes (Mono) and lymphocyte (Lymph) levels in mice from each experimental group (n=5 per group). Statistical analysis was performed with one-way ANOVA with Tukey multiple-comparison tests. *p<0.05, **p<0.01, ***p<0.001, ****p<0.0001.

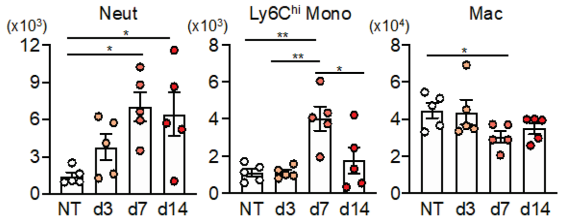

Supplemental Figure 6. Neutrophils infiltrate to the heart after Dox treatment in *wild type* mice. Flow cytometry analysis of cardiac immune cells from C57BL6/J wild type mice after Dox treatment (single injection of 15 µg/g) at different time points (0, 3, 7 ,14 days post injection respectively, n=5 per group). NT is “non-treated”. Statistical analysis was performed with one-way ANOVA with Tukey multiple-comparison tests. Neutrophil (Neut), monocyte (Mono) and macrophage (Mac).

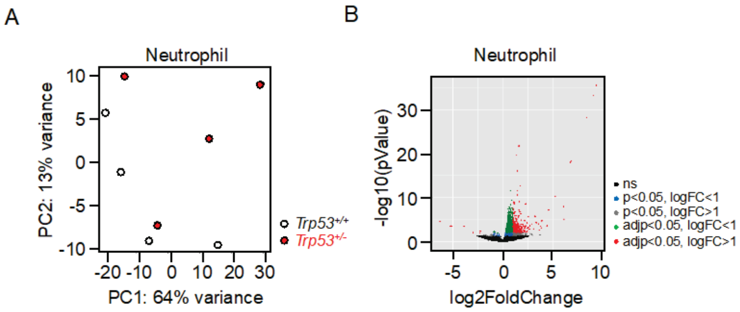

Supplemental Figure 7. *Trp53*-deficient neutrophils display a distinct gene expression profile. A. Principal component analysis (PCA) of ultra-low input RNA sequencing data obtained from sorted donor bone marrow-derived, peripheral blood neutrophils of *Trp53*-sufficient mice and *Trp53*- heterozygous deficient mice at 1 day after Dox injection (n=4 per genotype). **B.** Volcano plots showing the number of genes differently expressed between peripheral blood neutrophil from *Trp53*-sufficient vs. *Trp53*-heterozygous deficient mice at 1 day after Dox injection.

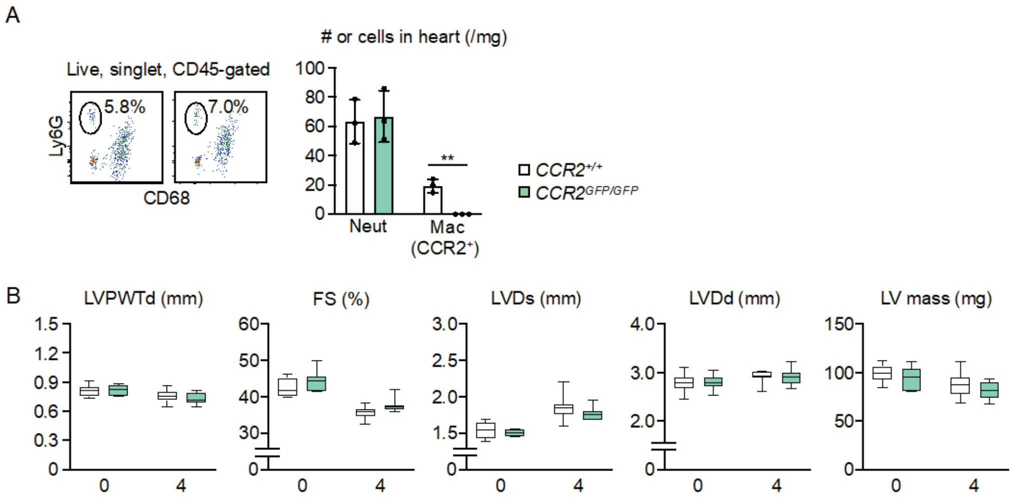

Supplemental Figure 8. Negligible contribution of monocytes to Dox-induced cardiotoxicity in wild type mice. A. Flow cytometry analysis of cardiac immune cells from *wild type* (*CCR2*$^{+/+}$) and *CCR2*-knockout (*CCR2*$^{GFP/GFP}$) mice 3 days after Dox administration (single injection of 15 mg/kg). Statistical analysis was performed with 2-tailed unpaired Student's *t* test (n=3 per genotype). Cell populations are defined as: Neutrophil=CD45$^+$Ly6G$^+$, infiltrating-monocyte/macrophage=CD45$^+$CD68$^+$CCR2$^+$. **B.** Echocardiographic analyses of left ventricular posterior wall thickness diameter (LVPWTd, mm), fractional shortening (FS, %), left ventricular end-systolic diameter (LVDs, mm), left ventricular end-diastolic diameter (LVDd, mm), and left ventricular mass (LV mass, mg) of *wild type* and *CCR2*-knockout mice at baseline and 4 weeks after Dox injection with a dose of 15 µg/g (body weight) split into 3 injections over 10 days (n=7-9 in each group). Statistical analysis was performed with 2-way repeated-measures ANOVA with Sidak's multiple-comparisons tests.

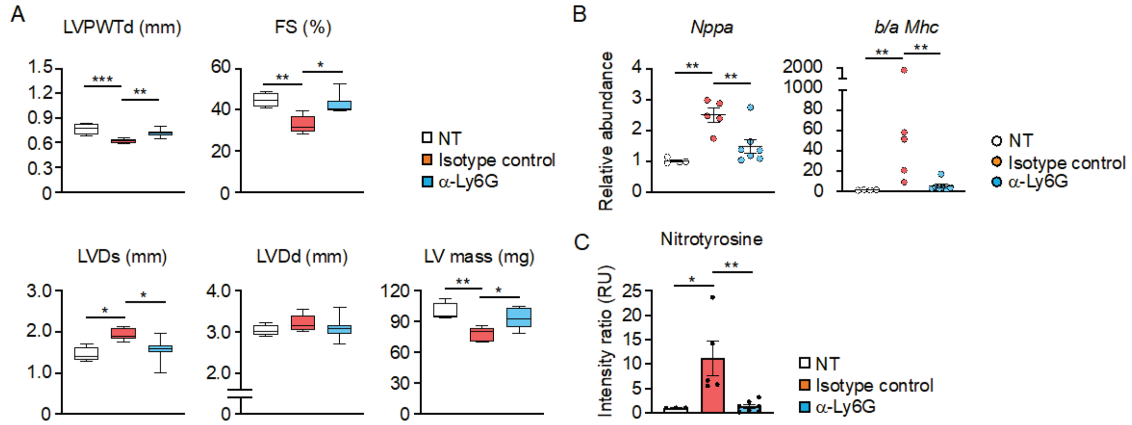

Supplemental Figure 9. Neutrophil depletion inhibits Dox-induced cardiotoxicity in wild type mice. A. Echocardiographic analysis of left ventricular posterior wall thickness diameter (LVPWTd, mm), fractional shortening (FS, %), left ventricular end-systolic diameter (LVDs, mm), left ventricular end-diastolic diameter (LVDd, mm), and left ventricular mass (LV mass, mg) of C57BL6/J mice at 2 weeks after treatment with sterile saline, or Dox + isotype control, or Dox + anti-Ly6G. 15 μg/g Dox (split into 3 injections) was intraperitoneally administered over a period of 10 days. The antibody was administered via intraperitoneal injection every 3 days (500 μg/injection/mouse) starting 2 days prior to Dox administration and continued until end of the study. Statistical analysis was performed with 1-way ANOVA with Tukey's multiple-comparisons tests (LVPWTd, LVDs, LVDd, LV mass) and Kruskal-Wallis H test with Dunn's multiple-comparisons tests (FS). **B**. Real time qPCR analysis of transcript expression in heart tissues of mice from each experimental group. *36b4* was used as a reference control. Statistical analysis was performed with Kruskal-Wallis H test with Dunn's multiple-comparisons tests. **C**. Relative immunofluorescence values of 3-nitrotyrosine staining in heart sections from the different experimental groups. Statistical analysis was performed with 1-way ANOVA with Tukey's multiple-comparisons tests. For A-C, n=4-7 in each group. **p*<0.05, ***p*<0.01, ****p*<0.001.

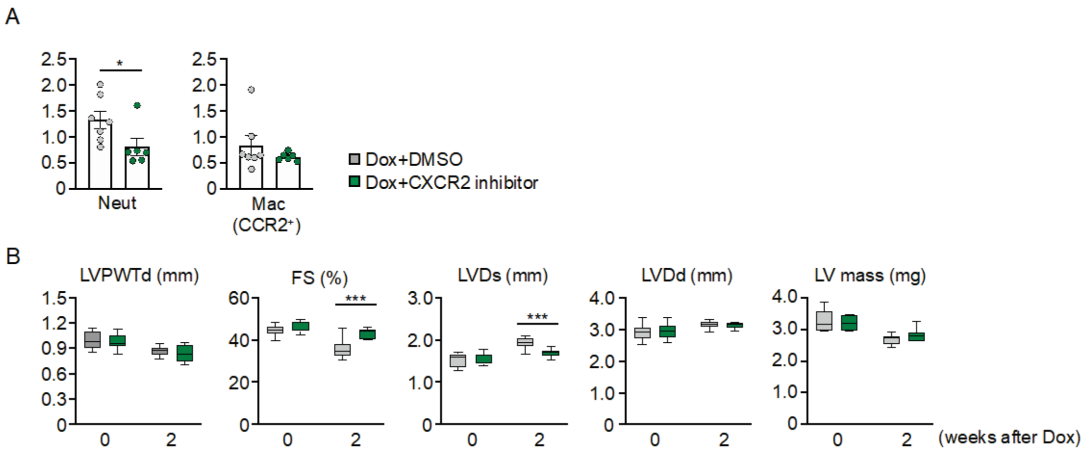

Supplemental Figure 10. A neutrophil recruitment inhibitor diminishes Dox-induced cardiotoxicity in wild type mice. A. C57BL/6J mice received Dox via an intraperitoneal injection (15 mg/kg split into 3 injections over 10 days) in the presence or absence of the CXCR2 inhibitor SB265610. The CXCR2 inhibitor was administered at a dose of 2 mg/kg/day via intraperitoneal injection initiating 1 day prior to Dox administration and continuing until the end of study. Control mice received an equivalent volume of the DMSO solvent for the SB265610. Flow cytometry analysis of cardiac immune cells from the hearts harvested at the end of study. Statistical analysis was performed with 2-tailed unpaired Student's *t* test (n=3 per group). **B.** Echocardiographic analyses of left ventricular posterior wall thickness diameter (LVPWTd, mm), fractional shortening (FS, %), left ventricular end-systolic diameter (LVDs, mm), left ventricular end-diastolic diameter (LVDd, mm), and left ventricular mass (LV mass, mg) of mice were determined at the 14-day timepoint. n=6-7 per group. Statistical analysis was performed with 2-way repeated-measures ANOVA with Sidak's multiple-comparisons tests. *$p<0.05$, ***$p<0.001$.

Supplemental Table 1. Total reads after adaptor trimming and Kallisto alignment.

	Sample ID	Total processed reads	%aligned
Trp53$^{+/+}$	4NWT1	75436734	84.5
	15LWT2	71971706	84.0
	17NWT3	82025052	84.8
	18RWT4	67990859	84.6
Trp53$^{+/-}$	13NHET1	105279399	83.1
	14RHET2	104355465	83.5
	19LHET3	96745097	86.1
	20RLHET4	89059415	80.4

Supplemental Table 2. Statistical information related to over-representation pathway analysis in *Trp53*-sufficient and *Trp53*-heterozygous insufficient blood neutrophils after Dox administration. Top significant Gene Ontology (GO) biological terms related to upregulated 898 genes and downregulated 63 genes in the *Trp53*$^{+/-}$ blood neutrophils after Dox administration. Number of genes, p value, and FDR are shown.

**Enriched in *Trp53*-insufficient neutrophils**	**No. of genes**	**p Value**	**FDR**
Immune response	75	<0.05	<0.05
Defense response	64	<0.05	<0.05
Response to cytokine	54	<0.05	<0.05
Cell morphogenesis involved in neuron differentiation	35	<0.05	<0.05
Regulation of developmental growth	26	<0.05	<0.05

**Enriched in *Trp53*-sufficient neutrophils**	**No. of genes**	**p Value**	**FDR**
Cell-cell adhesion	8	<0.05	above 0.05
Positive regulation of developmental process	11	<0.05	above 0.05

Supplemental Table 3. qPCR primer sequences used in this study.

Gene Name	Species	Forward	Reverse
Nppa	*Mus musculus*	5’- AAGAACCTGCTAGACCACCTG -3’	5’- TGCTTCCTCAGTCTGCTCAC -3’
aMHC	*Mus musculus*	5’- GCAGCAGCCCAGTACCTC -3’	5’- GTCATCAGGCACGAAGCA -3’
bMHC	*Mus musculus*	5’- CCTCACATCTTCTCCATCTCTG -3’	5’- TTGGATGACCCTCTTAGTGTTG -3’
Il1b	*Mus musculus*	5'- TGACAGTGATGAGAATGACCTGTTC -3'	5'- TTGGAAGCAGCCCTTCATCT -3'
Il6	*Mus musculus*	5'- GCTACCAAACTGGATATAATCAGGA -3'	5'- CCAGGTAGCTATGGTACTCCAGAA -3'
Tnf	*Mus musculus*	5’- CGGAGTCCGGCAGG -3'	5’- GCTGGGTAGAGAATGGATGAA -3'
36b4	*Mus musculus*	5'- GCTCCAAGCAGATGCAGCA -3'	5'- CCGGATGTGAGGCAGCAG -3'

Online Methods

SB265610 (CXCR2 inhibitor) administration

SB265610 is used to inhibit neutrophil infiltration into tissues. Mice were injected intraperitoneally with 2 mg/kg of SB265610, once a day, over a time course that starts 1 day before the start of doxorubicin administration until the end of the study. SB265610 was purchased from Sigma (Cat# SML0421). The solution of SB265610 was prepared in sterile dimethyl sulfoxide (DMSO) to a concentration of 10 mg/ml. The aliquoted mixture was stored at -20°C and thawed before use. The solution was mixed with corn oil (Sigma Cat# C8267) to minimize the toxic effect of DMSO just before injection. Injections were performed using a sterile syringe and needle (29G). The expiration date of the mixture coincided with the expiration date of the SB265610.

Echocardiography

Cardiac function was assessed before and after Dox administration at indicated time points using Vevo 2100 ultrasound system equipped with MS400D probe (VisualSonics, Fujifilm). Echocardiography was performed as described previously(28). Briefly, mice were anesthetized with isoflurane at a concentration of 5% (induction phase) and 1-1.5% (maintenance phase). Each animal was placed on the heating table in a supine position with the extremities taped to the table through four electrocardiography leads. Chest fur was removed with a chemical hair remover, and ultrasound gel was applied to the thorax surface to improve the visibility of the cardiac chambers. LVDd (left ventricular diameter at end-diastole, mm), LVDs (left ventricular diameter at end-systole, mm) (posterior wall thickness in diastole (PWTd), mm), and fractional shortening (FS, %) were measured from M-mode images obtained by short-axis view visualizing both mid papillary muscles. Measurements and analysis were performed by two individuals who were blinded to the identity of the experimental groups of mice using a Vevo Lab software (VisualSonics, Fujifilm).

Hematopoietic cell parameter measurements

Peripheral blood cells were obtained from the retro-orbital vein and collected in EDTA-coated tubes (BD). Hematopoietic parameters were analyzed using an Element HT5 Veterinary Hematology Analyzer (Heska, Loveland, CO).

Flow Cytometry

Flow cytometry analysis of bone marrow cells, peripheral blood cells, and cardiac immune cells were performed as reported in our previous studies. The antibodies used for flow cytometric analysis are as described in our previous publications (14, 28, 39)**.** Bone marrow: Bone marrow cells were flushed out from 1 femur and 1 tibia, and red blood cells were lysed with RBC lysis buffer (BioLegend, Cat# 420301) for 20 seconds at room temperature. Dead cell staining was performed using Zombie Aqua™ Fixable Viability Kit (BioLegend, Cat# 423102) in PBS for 15 minutes at room temperature. Cells were labeled with biotin-conjugated anti-mouse antibodies, and followed by incubation with BV650-Streptavidin (BioLegend, Cat# 405231) together with fluorochrome-conjugated antibodies for 20 minutes respectively at room temperature. Peripheral blood: Peripheral blood cells were obtained from the retro-orbital vein. Red blood cells were lysed with eBioscience™ 1X RBC Lysis Buffer (Thermo Fisher, Cat# 00-4333- 57) for 5 minutes on ice. Incubation with antibodies was done for 20 minutes at room temperature in the dark. Heart tissue: Hearts were flushed with 15 ml of cold PBS from apex, and right atrium was excised to facilitate perfusion. Right ventricles and atriums were removed, and left ventricles were minced and digested in an enzyme cocktail: collagenase I (450 U/ml), collagenase XI (125 U/ml), Hyaluronidase (450 U/ml), and DNase I (60 U/ml) (Cat# C0130, C7657, H3506, and D4513, respectively, Sigma-Aldrich), with the setting of 900 rpm, 37°C, 30 minutes in ThermoMixer® C (Eppendorf). Hearts were subsequently homogenized through a 70 μm cell strainer (Fisher Scientific, Cat# 352350).

Viable cells were identified as unstained cells with Zombie Aqua. Incubation with antibodies was done for 20 minutes at room temperature in the dark. 123count eBeads (Thermo Fisher, Cat# 01-1234-42) were used for quantification of absolute cell numbers. BD LSRII Flow Cytometer or Fortessa (BD Bioscience) was used for data acquisition. Data were analyzed with FlowJo Software.

Quantitative RT-PCR

Mouse total RNA was isolated using QIAzol reagent (Qiagen, Cat# 79306), and was purified on the QIAcube. 1 µg RNA was reverse transcribed with High-capacity cDNA Reverse Transcription Kit (Thermo Fisher Scientific, Cat# 4368814). qRT- PCR was performed with PowerUp SYBR® Green reagent (Thermo Fisher Scientific, Cat# 4367659) in a QuantStudio 6 Flex PCR system. Primers for gene expression studies are shown in the **Supplemental Table 3**. Gene expression was calculated relative to *36b4* and normalized to controls. Data were calculated by $\Delta\Delta$Ct approach.

Histology

Nitrotyrosine staining of hearts

Mice were sacrificed and perfused with cold PBS from the left ventricular apex. Hearts were excised and fixed with 10% formalin at 4 °C overnight. Heart tissues were dehydrated, embedded in paraffin and sectioned at a thickness of 7µm. All histological analysis was performed with sections at the level of the papillary muscles. Nitrotyrosine staining was performed as follows. After deparaffinization with xylene and rehydration through decreasing ethanol concentrations, slides were heated at 98°C to induce antigen retrieval with citrate buffer (pH 6.0) (Abcam#93678). After three wash with TBS plus 0.05% Tween 20 (TBS-T), slides were incubated for 1 hour with 5% normal goat serum (#S26, EMD-Millipore) to block non-specific antibody binding. Then,

sections were incubated for 2 hours with AffiniPure Fab Fragment Goat Anti-Mouse IgG (H+L) (Jackson Immuno Research Lab, #115-007-003) to inhibit endogenous mouse immunoglobulins. Then, slides were incubated overnight at 4℃ with mouse anti-nitrotyrosine antibody (1:100; #sc-32757; Santa Cruz Biotechnology) following by 1 hour incubation with Alexa Fluor 488 goat anti-mouse secondary antibody (#11001; Thermo Fisher Scientific). Sections were also counterstained with DAPI (#D1306, Thermo Fisher) and images were captured at 20× using a "all in one" BZ-X710 fluorescent microscope (Keyence Corp). Immunofluorescence staining was quantified by using the Fiji-ImageJ software and the arithmetic mean of six images was calculated for each sample. As a negative control, hearts from doxorubicin treated mice were incubated with blocking buffer and second antibody, but not with the primary antibody.

Picro Sirius/Fast Green staining of heart

Deparaffinized sections were stained with freshly prepared PBS-based staining buffer composed of 1.2%/w saturated aqueous picric acid, 0.1%/w Fast Green FCF and 0.1%/w Direct Red 80 (Cat# 197378, Cat# F7252, Cat# 365548, respectively, from Sigma-Aldrich). Sections were incubated for 1 h at room temperature and the images were analyzed by Fiji-image J software for quantification of collagen content. Myocardial fibrosis size was expressed as a percentage of total LV area.

Myocyte cross-sectional area (CSA)

To determine CSA, wheat germ agglutinin staining of the heart sections from each experimental group was performed using Alexa Fluor 594 conjugated-WGA (Life Technologies, Cat# W11262). Quantification of cardiomyocyte CSA was performed by an operator which is blinded to mouse genotypes and used a computer-assisted morphometric analysis tool on a Keyence BZ-X710 microscope. On average, 80-100 round-shaped cardiomyocytes per section were randomly selected for CSA analysis.

Capillary density measurement

Capillary density was quantified by staining with Alexa Fluor 488-conjugated GS-Isolectin IB4 (Invitrogen, Cat# I21411). Exact numbers of cardiomyocytes and capillaries in the same view were measured and the ratio #capillaries/#cardiomyocytes was calculated as indicative of capillary density. On average, 5 views per section were randomly selected for capillary density analysis.

Ultra-low input RNA sequencing

For RNA sequencing analysis of blood neutrophil (CD45.2^{+}Ly6G^{+}CD115^{-}), mice peripheral blood was initially collected in EDTA-coated tubes. After lysis of red blood cells, cells were stained with CD45.2, Ly6G, CD115 monoclonal antibodies. DAPI was used to exclude dead cells. Sorting was performed on FACS Aria Fusion Cell Sorter platform with an 85μm nozzle and flow pressure set to 45 psi. A total of 50,000 cells were sorted directly into 1ml of Trizol (Qiagen). Samples were sent to Genewiz and subjected to library construction and sequencing. The HiSeq4000 platform was used for sequencing to generate paired end data of 150bp. Sequence alignment and quantitation was performed by Kallisto 0.44.0. *Mus musculus* GRCm38 FASTA file from Ensembl database was used as an index for alignment. Differential gene expression analysis was performed using different R-studio analysis packages. WebGestalt online tool was used for the identification of statistically overrepresented (enriched) pathways among the differentially expressed genes identified by RNA-Seq analysis. After submission of the gene list, overrepresentation analysis on biological processes was performed based on GO with the *Mus musculus* genome. To identify the most significantly enriched gene sets, redundancy reduction was done with "weighted set cover" method.
